# MetaCluster-TA: taxonomic annotation for metagenomic data based on assembly-assisted binning

**DOI:** 10.1186/1471-2164-15-S1-S12

**Published:** 2014-01-24

**Authors:** Yi Wang, Henry Chi Ming Leung, Siu Ming Yiu, Francis Yuk Lun Chin

**Affiliations:** Department of Computer Science, The University of Hong Kong, Kragujevac, Hong Kong

## Abstract

**Background:**

Taxonomic annotation of reads is an important problem in metagenomic analysis. Existing annotation tools, which rely on the approach of aligning each read to the taxonomic structure, are unable to annotate many reads efficiently and accurately as reads (~100 bp) are short and most of them come from unknown genomes. Previous work has suggested assembling the reads to make longer contigs before annotation. More reads/contigs can be annotated as a longer contig (in Kbp) can be aligned to a taxon even if it is from an unknown species as long as it contains a conserved region of that taxon. Unfortunately existing metagenomic assembly tools are not mature enough to produce long enough contigs. Binning tries to group reads/contigs of similar species together. Intuitively, reads in the same group (cluster) should be annotated to the same taxon and these reads altogether should cover a significant portion of the genome alleviating the problem of short contigs if the quality of binning is high. However, no existing work has tried to use binning results to help solve the annotation problem. This work explores this direction.

**Results:**

In this paper, we describe MetaCluster-TA, an assembly-assisted binning-based annotation tool which relies on an innovative idea of annotating binned reads instead of aligning each read or contig to the taxonomic structure separately. We propose the novel concept of the 'virtual contig' (which can be up to 10 Kb in length) to represent a set of reads and then represent each cluster as a set of 'virtual contigs' (which together can be total up to 1 Mb in length) for annotation. MetaCluster-TA can outperform widely-used MEGAN4 and can annotate (1) more reads since the virtual contigs are much longer; (2) more accurately since each cluster of long virtual contigs contains global information of the sampled genome which tends to be more accurate than short reads or assembled contigs which contain only local information of the genome; and (3) more efficiently since there are much fewer long virtual contigs to align than short reads. MetaCluster-TA outperforms MetaCluster 5.0 as a binning tool since binning itself can be more sensitive and precise given long virtual contigs and the binning results can be improved using the reference taxonomic database.

**Conclusions:**

MetaCluster-TA can outperform widely-used MEGAN4 and can annotate more reads with higher accuracy and higher efficiency. It also outperforms MetaCluster 5.0 as a binning tool.

## Background

Text for this section. Metagenomics is the study of an entire community of microorganisms from an environmental sample. High-throughput next-generation sequencing (NGS) provides an opportunity to sequence and analyze genomes of multiple species from an environmental sample without cultivation. During the last several years, researchers have done many successful metagenomic projects on different samples based on NGS, such as human gut [1, 2] and cow rumen [3]. One of the important functions in metagenomic NGS analysis is to annotate to what species or what taxonomic group the metagenomic data belongs. This provides information on what kinds of species exist in the sample for further downstream analysis.

There are two existing fundamental types of tools for metagenomic data analysis, namely *assembly* and *binning*. Assembly tools try to reconstruct the genomes that exist in the sample. Binning tools try to group the NGS reads of similar species together.

In the ideal case, if we can assemble each species in the metagenomic sample, we can solve the annotation problem relatively easily. However, existing assembly tools are far from the ideal case and assembling metagenomic data is still a challenging and unresolved problem, although metagenomic assemblers can construct longer contigs.

On the other hand, advances have been made for binning. Existing binning strategies can be divided into two categories: *supervised* methods (also called similarity-based methods) and *unsupervised* methods (also called composition-based methods).

Supervised binning methods [4, 5] are the most common approaches for analyzing metagenomic samples. They make use of known genomes and sequence similarities among reads or contigs (after assembly). Some supervised methods use generic features, such as 16S rRNA small subunit, recA and rpoB, to classify fragments. However, a large percentage (> 99%) of reads (or contigs) do not have these features [6]. Moreover, one species may have multiple markers and multiple species may share the same marker [7].

Unsupervised methods, which do not rely on known genome information, are usually group reads/contigs together based on three observations: (A) the *k-*mer frequency of reads, where *k* ≅ 16, is generally linearly proportional to the abundance of the corresponding species [8]; (B) sufficiently long *w*-mers, where *w* ≥ 36, have very high probability to be unique in each species [9]; and (C) sufficiently long reads/contigs from the same or similar species tend to have similar short *q*-mer distributions, where *q* = 4 or 5, [10–15].

AbundanceBin [8], which is based on Observation (A), cannot separate reads from species with similar abundance. A recent tool, Improved-TOSS [16], uses Observation (A) to group reads together if they are from species with similar abundance, and then uses Observation (B) to separate reads from different species for each group. Improved-TOSS has good sensitivity performance for small datasets. MetaCluster 4.0 [11] is composed of three phases: Phase 1 groups reads according to a probabilistic model based on Observation (B); Phase 2 derives *q*-mer distribution; and Phase 3 further merges groups together with *K*-means clustering based on Observation (C). MetaCluster 5.0 [14] uses an extra round whose approach is based on Observation (A) to handle species of extremely low abundance in noisy samples. The MetaCluster software solves some important issues in unsupervised binning methods such as processing large datasets with many species and dealing with species of different abundance. When the number of species increases (e.g. for the largest testing dataset T7 in [16]), MetaCluster 5.0 achieves better precision and sensitivity.

Despite the recent advances in binning, to solve the annotation problem, we still rely on the approach of aligning each read to the taxonomic structure [17, 18]. In particular, MEGAN4 [18], which is widely used in metagenomic analysis, is based on this approach. Common annotation approaches, like MEGAN4, can be classified as 'nearest neighbor' methods [19], as they usually assign reads to the lowest common ancestor (LCA) from the taxonomy of most similar sequences in the database. If the read can only be aligned to a single genome in the database, the read will be annotated to the species/subspecies of that genome. If the read can be aligned to many genomes, depending whether these genomes are within a species/genus/family, the read will be annotated to that species/genus/family.

This procedure is time consuming and many reads cannot be aligned to any known sequences because many sequences for microorganisms remain unknown [20]. For better results, contigs after assembly, instead of reads, are used for annotation [21]. As the reads contributing to a contig are likely to belong to a single genome, using contigs for annotation has several advantages: (a) contigs can be aligned and annotated to a genome more readily than reads because contigs (of Kbp length) are much longer than reads, and (b) annotating a contig is equivalent to annotating all reads contributing to this contig, even though some of these reads cannot be aligned individually.

To summarize, this approach of annotating metagenomic data by aligning each read/contig to the reference genomes in the taxonomic structure has the following shortcomings.

1) *Unable to annotate many reads* - Methods that rely on alignment of reads/contigs to known genomes still fail to align a large number of reads if they are from unknown species. Failure to align means that the read cannot be annotated. Contigs which are longer can be used instead of reads [21] as they can be aligned to known genomes with higher confidence and thus, more reads which are associated with the contigs can be annotated. But the improvement is limited due to the limited success in the assembly problem for producing long contigs.

2) *Less precise annotation for reads and more incorrect annotation for contigs* - Even though the reads/contigs are from a particular species, they may be aligned to similar but different species under the same genus or family and thus be assigned to a higher taxonomy level. This means less precise in annotation. This problem can be slightly alleviated for contigs as contigs are longer and the alignment can be more precise, thus resulting in a more precise annotation. However, since contigs are still short when compared with the length of a genome and can only capture some local information of the unknown genome, the problem of imprecise annotation cannot be solved completely. Even worse, there are cases that these contigs can be easily aligned to multiple genomes locally (due to horizontal gene transfer or housekeeping genes, etc) that make the annotation incorrect.

3) *Inefficient or time-consuming annotation* - Annotating reads/contigs based on genomes of known species in the database may take a long time when certain errors are allowed during alignment. Even when the reads are assembled into contigs, there are still many contigs to align and annotate.

### Our contributions

In this paper, we extend the simple idea that contigs have better annotation performance than reads by using binning results, i.e., clusters, to perform annotation. Assembly is used to produce longer contigs which help annotation, but also better binning results indirectly. We try to align each cluster to a taxon. To achieve this, we introduce the novel concept of *virtual contigs*, which are longer than traditional contigs because they are not, strictly speaking, contigs but functionally help to connect related reads together as a single unit for binning purposes, e.g. reads/contigs are binned together by paired-end reads or substantially-overlapped regions. Thanks to long virtual contigs, clustering by means of *q*-mer distribution can be more sensitive and precise since 5-mers for contigs of length longer than 10 k bp can be used to yield better clustering results than the 4-mers used in MetaCluster 5.0. Note that contigs/virtual contigs can only contain local information of a genome. However, reads/contigs merged into a cluster through *q*-mer distribution can be far apart and thus can capture some global information of a genome. Unsupervised binning can cluster together reads from unknown species and such a cluster could be potentially annotated by the species/genus/family to which many of the reads/contigs in the cluster belong.

The introduction of the virtual contig and binning techniques, which produces better clustering results for annotation, has further benefits:

1. *More annotated reads* - An otherwise-unaligned read can be binned and annotated together with the other reads of the virtual contig (not only contigs) to which it belongs.
2. *More accurate annotation* - The virtual contigs, which cover longer regions, can be better aligned to the genome of a particular species, reducing the likelihood that reads would be inaccurately assigned to a higher taxonomy. Furthermore, reads in a cluster are annotated together through the information of reads/contigs/virtual contigs in the cluster. The annotation is more precise, because clusters are much larger in size (in terms of Mbp) and contain global information. The problem of horizontal gene transfers or housekeeping genes can be resolved because they only affect relative short regions (in terms of Kbp).
3. *More efficient annotation* - Efficiency can be gained by annotating fewer clusters, instead of many individual reads/contigs. The number of clusters is usually about the number of species in the dataset, which is far smaller than number of reads/contigs.

## Results

We compared the performance of MetaCluster-TA with MetaCluster 5.0 and MEGAN4, since MetaCluster 5.0 is the most advanced unsupervised binning tool and MEGAN4 is a widely-used supervised binning and annotation tool. All the experiments were run on a UNIX machine with 4CPU of Intel Xeon X5650@2.4GHz.

In practice, reads from genomes of known species in the database can be annotated easily by alignments, e.g., BLASTN in MEGAN4. The main problem is those reads from unknown species. Reads from an unknown genome are usually annotated according to their similarity (by alignment) to the known genomes. In order to simulate the metagenomic environment of unknown species, instead of using the NCBI complete genome database (http://ftp.ncbi.nih.gov/genomes/), a set of genomes, called *target genomes*, which represent the set of unknown species, were selected and removed from the database. At the same time, we have to ensure that genomes of some related/similar species exist in the database (*reference genomes*). We say that the set of reads/contigs of an unknown (target) genome has *species-reference* if there exists at least one reference genome of the same species in the database as the unknown genome. Similarly, if there exists at least one reference genome in the database from the same order as the reads/contigs of the target genome and there does not exist any reference genome in the database that belongs to a lower taxonomy level of that order, i.e., same as the target genome's species, genus or family level, we say that these reads/contigs have *order-reference*.

In our experiments, the testing datasets were generated from NCBI complete genome database (http://ftp.ncbi.nih.gov/genomes/). Based on the set of target genomes, we randomly generated a set of length-75 paired-end reads with 1% sequencing error and 250 ± 50 bp insertion distances according to some specified abundance. Three datasets, A1, A2 and B, were generated and detailed information about these three datasets such as number of selected genomes, selected genome names, coverage and reference genomes used in our experiments can be found on our website http://i.cs.hku.hk/~alse/MetaCluster/files/Datalist_Of_MetaCluster-TA.

### Improvement on annotation

One important advantage of this method is its effectiveness in annotating more species. To evaluate the performance, we generated two testing datasets, one with high coverage (A1) and the other with low coverage (A2).

A total of 50 target genomes were picked from different species to generate testing data. In addition to the removal of these 50 genomes from the reference database for simulating the scenario of the unknown species, we also remove genomes from the reference database so that reads from 25 genomes have species-reference (all genomes from the same subspecies as the target genome are removed) and the other 25 genomes have order-reference only (all genomes from the same family as the target genome are removed). Reads sampled from these 50 genomes are used to generate the two datasets A1 and A2. High coverage dataset A1 is generated by sampling reads from the 50 species with coverage of about 15×. Another low coverage dataset A2 is generated by sampling reads from the 50 species (two groups of 25 species, one group has specific reference and the other order-reference). In each group of 25 species, 20 are of coverage ≤ 3 and 5 are of coverage 8.

Two experiments on MEGAN4 were performed for each dataset, one on metagenomic reads directly and the other on contigs after assembly using IDBA-UD [22]. As all reads contributing to the contig will be annotated with the taxonomy of the contig, more reads might be annotated. Reads that cannot be assembled will be treated as a single contig for annotation. In this section, we will compare MetaCluster-TA with MEGAN4 on these two approaches, namely MEGAN4 (reads) and MEGAN4 (contigs), on two datasets, A1 and A2. As MEGAN4 takes BLASTN results as inputs, default parameters were used to run BLASTN and MEGAN4. Annotation performances on datasets A1 and A2 are shown in Table 1 and Table 2 respectively.

**Table 1:** Annotation result on high-coverage dataset A1

Methods	Species-reference (~16.7 million reads)				Order-reference (~20.0 million reads)			
	**Accurate**	**Higher**	**Incorrect**	**Unassigned**	**Accurate**	**Higher**	**Incorrect**	**Unassigned**
MetaCluster-TA	**60.9%**	**32.9%**	**4.0%**	**2.2%**	**31.8%**	**38.1%**	22.6%	**7.5%**
MEGAN4 (contigs)	60.7%	12.9%	22.3%	4.1%	12.3%	13.1%	65.3%	9.3%
MEGAN4 (reads)	57.7%	14.8%	4.6%	22.8%	0.7%	0.7%	**3.4%**	95.2%

* "Accurate" corresponds to the percentage of species-reference/order-reference reads annotated correctly, i.e., their correct species/order names of the target genomes; "Higher" corresponds to the percentage of species-reference/order-reference reads that are correctly annotated, but to taxonomy of higher levels than species/order of the target genomes (e.g. reads of *E. coli*-reference annotated with family name *Enterobacteriaceae*); "Incorrect" corresponds to the percentage of reads which are annotated incorrectly; "Unassigned" corresponds to the percentage of reads that cannot be annotated to any taxonomy.

* Running time of MetaCluster-TA is about 8 hours; running time of MEGAN4 (reads) is about 4 days; running time of MEGAN4 (contigs) is about 1 day.

* About 80% reads can be aligned to contigs of length > 500 bp with <5% mismatches.

**Table 2:** Further comparison between MEGAN4 (contigs) and MetaCluster-TA on species-reference of A1

		MetaCluster-TA			
		**Accurate**	**Higher**	**Incorrect**	**Unassigned**
MEGAN4 (contigs)	Accurate	56.3%	3.1%	1.3%	0.0%
	Higher	3.2%	8.4%	1.3%	0.0%
	Incorrect	1.3%	19.6%	1.4%	0.0%
	Unassigned	0.1%	1.8%	0.0%	2.2%

We compared the performance of the annotation algorithms based on four aspects: "Accurate" annotation, "Higher" annotation, "Incorrect" annotation and "Unassigned" reads. "Accurate" annotation refers to the reads annotated to the correct taxonomy, i.e. reads sampled from species-reference and order-reference genome annotated to the correct species level and order level respectively. "Higher" annotation refers to the reads annotated correctly but to higher taxonomy than the target genome, e.g. reads sampled from species-reference target genome are annotated to the family or higher taxonomy level of the target genome; similarly, reads sampled from order-reference target genome are annotated to the class or higher taxonomy level of the target genome. "Higher" annotation is considered as a correct but less precise annotation. "Incorrect" annotation refers to the reads annotated to wrong taxonomy. "Unassigned" reads are the reads that cannot be annotated by the corresponding software. Since a read can be annotated "Accurate", "Higher", "Incorrect" or "Unassigned", the sum of the percentages in the corresponding part of each row in Table 1 and 2 should be 100%.

Assume read *R* from target genome *G* is annotated with taxonomy *T*. We say *R* is *correctly annotated* if *G* is in taxonomy *T* (i.e., *R* is in the category "Accurate" or "Higher"). An annotation tool with good performance would be able to correctly annotate more reads, has less incorrect annotation and have less unassigned reads, i.e. more "Accurate" or "Higher" and less "Incorrect" or "Unassigned" reads.

For reads with species-reference in the high-coverage dataset A1, MetaCluster-TA has about 20% more "Higher" reads and slightly more "Accurate" reads than MEGAN4 (for both reads and contigs input). Since the target and the reference genomes are similar, all methods have high and similar "Accurate" annotations as many reads are from their common regions and are aligned to reference genomes correctly. For those reads from not-so-similar regions, they can only be annotated as a group together in a cluster and contig with more information. Thus, MetaCluster-TA and MEGAN4 (contigs) have more assigned reads. MEGAN4 (reads) has about 20% more unassigned reads than the other two. According to Table 1, MEGAN4 (contigs) does not have more "Higher" annotation as expected because the BLASTN alignment algorithm based on the default parameters might only depend on the matching of some short patterns in the contigs and this might result in the wrong annotation. MetaCluster-TA generates much longer sequences to do alignment, and the alignment result is supposed to be more accurate. Thus, this explains why even though MEGAN4 (contigs) can assign more reads than MEGAN4 (reads), it has about 20% more "Incorrect" reads than the other. As MEGAN4 (reads) annotates each read independently and cannot dig out taxonomic information for unaligned reads, MEGAN4 (reads) has the most "Unassigned" reads.

As for reads with order-reference, since the reference and target genomes are less similar, fewer reads will be aligned. Consider MEGAN4 (reads), 95.2% are "Unassigned" and only 0.7% are "Accurate" annotated. MetaCluster-TA and MEGAN4 (contigs) have more "Accurate" annotations in the order that the former has more information (global) than the latter (local) for alignment; similar arguments for explaining why the numbers of "Unassigned" annotation are in the reverse order. Note that MEGAN4 (contigs) has the largest number of "Incorrect" annotation score with more serious errors (than species-reference) partially because of the previous explanation about the BLASTN alignment algorithm and because the genomes are not-so-similar which leads to incorrect annotations. Specifically when compared to MEGAN4, MetaCluster-TA has much less "Incorrect" or "Unassigned" reads, about 20% more "Accurate" reads and about 25% more "Higher" reads. Note that the incorrect percentage is based on all reads. If we only consider the annotated reads, the incorrect percentage for MEGAN4 (reads) will be much higher since it annotates much fewer reads, thus the precision of MEGAN4 (reads) is not high.

Table 2 and 3 make further comparisons between MEGAN4 (contigs) and MetaCluster-TA on species-reference reads and order-reference reads respectively. For the species-references data, since most of the contigs and reads can be aligned to the reference genomes well, it is expected that there is not much improvement by MetaCluster-TA. However, among the reads annotated 'Incorrect' by MEGAN4 (contigs), MetaCluster-TA successfully annotated 19.6% to 'Higher'. It is because the cluster contains global information of the set of contigs and reads and prevents incorrect annotation due to local similarity between different species. When there is no similar genome as reference, i.e. the order-reference data, MEGAN4 (contigs) have more incorrect annotation and MetaCluster-TA can correct 20.5% reads to 'Accurate' and 27.2% reads to 'Higher'.

**Table 3:** Further comparison between MEGAN4 (contigs) and MetaCluster-TA on order-reference of A1

		MetaCluster-TA			
		**Accurate**	**Higher**	**Incorrect**	**Unassigned**
MEGAN4 (contigs)	Accurate	9.3%	1.3%	1.7%	0.0%
	Higher	1.3%	8.7%	3.1%	0.0%
	Incorrect	20.5%	27.2%	17.6%	0.0%
	Unassigned	0.7%	0.9%	0.2%	7.5%

For the low-coverage dataset A2 (Table 4), MEGAN4 (reads) has similar performances to the high-coverage dataset in all situations as each read is aligned and annotated independently without taking coverage into consideration. MEGAN4 (contigs) can only have slightly better performances than MEGAN4 (reads) and have much lower "Incorrect" annotation because the coverage is too low for assembly. MetaCluster-TA has the best annotation performance in terms of the numbers (percentages) of "Accurate" and "Higher" reads because clusters can still be formed with low coverage reads as MetaCluster 5.0 can handle low coverage binning reasonably well. For reads with order-reference, MetaCluster-TA has >20% more "Accurate" reads and >10% more "Higher" reads than MEGAN4. Since species in A2 has low coverage, fewer and shorter contigs are formed and fewer reads can be associated with contigs. Thus, both MetaCluster-TA and MEGAN4 have more "Unassigned" reads for the low-coverage dataset than for the high-coverage dataset. Nevertheless, MetaCluster-TA has the least number of "Incorrect" and "Unassigned" reads.

**Table 4:** Annotation result on dataset A2

Methods	Species-reference (~16.7 million reads)				Order-reference (~20.0 million reads)			
	**Accurate**	**Higher**	**Incorrect**	**Unassigned**	**Accurate**	**Higher**	**Incorrect**	**Unassigned**
MetaCluster-TA	**58.7%**	**8.9%**	**3.8%**	**28.6%**	**35.7%**	**15.1%**	14.4%	**34.8%**
MEGAN4 (contigs)	54.2%	1.8%	3.8%	40.1%	13.5%	4.3%	42.0%	40.3%
MEGAN4 (reads)	57.3%	5.9%	4.3%	32.6%	1.1%	0.5%	**3.9%**	94.5%

*Running time of MetaCluster-TA is about 5 hours; running time of MEGAN4 (reads) is about 1.5 days; running time of MEGAN4 (contigs) is about 10 hours.

* About 40% reads can be aligned to contigs of length > 500 bp with <5% mismatches.

Table 5 and 6 show further comparison between MEGAN4 (contigs) and MetaCluster-TA on species-reference reads and order-reference reads respectively. The results are similar as in Table 2 and 3. Among the species-reference reads annotated 'Unassigned' by MEGAN4 (contigs), MetaCluster-TA annotated 4.3% to 'Accurate' and 6.3% to 'Higher'. Among the order-reference reads annotated 'Incorrect' by MEGAN4 (contigs), MetaCluster-TA annotated 21.3% to 'Accurate' and annotated 11.9% to 'Higher'.

**Table 5:** Further comparison between MEGAN4 (contigs) and MetaCluster-TA on species-reference of A2

		MetaCluster-TA			
		**Accurate**	**Higher**	**Incorrect**	**Unassigned**
MEGAN4 (contigs)	Accurate	51.1%	0.7%	2.4%	0.0%
	Higher	0.8%	1.0%	0.0%	0.0%
	Incorrect	2.5%	0.9%	0.4%	0.0%
	Unassigned	4.3%	6.3%	1.0%	28.6%

**Table 6:** Further comparison between MEGAN4 (contigs) and MetaCluster-TA on order-reference of A2

		MetaCluster-TA			
		**Accurate**	**Higher**	**Incorrect**	**Unassigned**
MEGAN4 (contigs)	Accurate	12.2%	1.0%	0.3%	0.0%
	Higher	1.6%	0.6%	2.1%	0.0%
	Incorrect	21.3%	11.9%	8.7%	0.0%
	Unassigned	0.6%	1.6%	3.3%	34.8%

Another important advantage is that MetaCluster-TA is much more efficient. MEGAN4 (reads) and MEGAN4 (contigs) take about 4 days and 1 day respectively to complete a run, while MetaCluster-TA takes about 8 hours, including the time for assembly.

### Improvement on clustering

Another important contribution of MetaCluster-TA is its clustering performance. In our hybrid approach, clustering takes advantage of taxonomy information.

Assume a binning method outputs *M* clusters *C*_*i*_ (1 ≤ *i* ≤ *M*) and there are *N* genomes in the sample. Let *R*_*ij*_ be the number of reads in *C*_*i*_ that belong to genome *j*. Cluster *C*_*j*_ represents genome *j*_0_ iff *R*_*ij*0_= max_*j*_*R*_*ij*_. Following the definition of precision and sensitivity of the clustering results as given in [14], we have:

MetaCluster 5.0 is designed to overcome binning difficulties like extremely-low/low-coverage species, uneven coverage, and dataset containing too many species. To make fair comparison with MetaCluster 5.0 on clustering, we generated testing dataset B with reads sampled from genomes with different coverages.

We randomly picked 100 species and selected one genome from each species. Their coverages vary from 1 to 20 and there are 5 genomes for each coverage. Thus, there are 55 species with ≥ 10× coverage, 20 species with [6×,10×) coverage and 25 species with <6× coverage.

Consider all reads sampled from a species *S*. If there exists a cluster *C* such that >50% reads sampled from *S* are in *C* and >50% reads in *C* are from *S*, we say that S is *discovered* by *C*.

The results are shown in Table 7. For high coverage species (>10×), these two methods discovered the same number of species (47 out of 55 species), but MetaCluster-TA has better sensitivity. For low coverage species [6×,10×), MetaCluster 5.0 discovered 11 out of 20, while MetaCluster-TA discovered 3 more. MetaCluster-TA also achieves higher sensitivity. Overall, hybrid approach gets 6% higher precision and 8% higher sensitivity. Since the hybrid approach needs to annotate clusters, MetaCluster-TA requires reasonably more running time.

**Table 7:** Clustering performance on dataset B

	Species discovered			Sensitivity		Overall performance			
	**≥10×**	**[6×, 10×)**	**<6×**	**≥10×**	**[6×, 10×)**	**Precision**	**Sensitivity**	**Memory**	**Time**
MetaCluster 5.0	47	11	0	0.84	0.78	0.80	0.79	35 GB	101 min
MetaCluster-TA	47	14	0	0.89	0.84	0.86	0.87	30 GB	250 min

## Discussion

For metagenomic projects, clustering and binning/annotation remain difficult problems. Existing methods consider these two processes separately. However, better binning results can assist better annotation and an accurate annotation can improve the quality of annotated clusters. By considering them together, hybrid methods may achieve better results for both clustering and binning.

## Conclusions

MetaCluster-TA can outperform widely-used MEGAN4 and can annotate more reads with higher accuracy and higher efficiency. It also outperforms MetaCluster 5.0 as a binning tool.

## Methods

**Figure 1:**
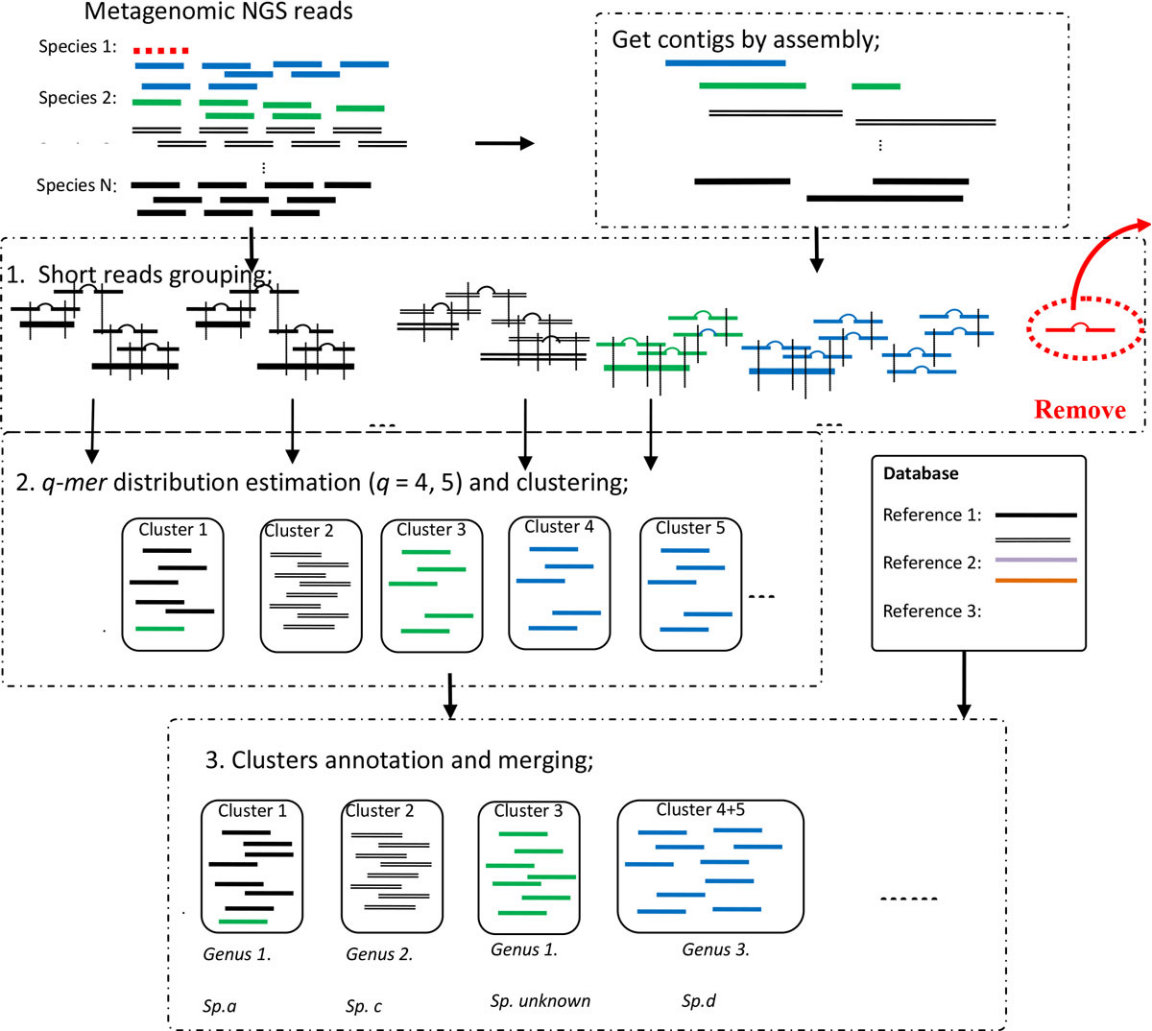
**Workflow of MetaCluster-TA**.

MetaCluster-TA is an assembly-assisted approach for the binning and annotation of metagenomic NGS reads. Instead of annotating each read or assembled contig separately, it bins similar reads/contigs into the same cluster and annotates the whole cluster. The annotation information could also be used for improving the clustering process and thus the annotation results. As shown in Figure 1, MetaCluster-TA consists of three phases: (1) construction of long virtual contigs from assembly and probabilistic grouping of short reads; (2) q-mer distribution estimation and clustering; (3) cluster annotation and merging. We will describe each phase in detail in the following sections.

### Phase 1: construction of long virtual contigs

MetaCluster-TA applies a similar method as MetaCluster 5.0 (based on Observation (B)) to group short reads into clusters, i.e. the probability of two clusters of reads sharing common *w*-mer (length-*w* substring with *w* > 35) being sampled from the same species are calculated [14] and the clusters are merged into a single cluster if the probability is high. However, instead of considering each read as an initial cluster, we assemble reads into long contigs (> 500 bp) and consider reads aligned to the same contig (> 95% matches) as the initial cluster. The idea is that reads aligned to the same contig have higher probability of being sampled from the same species because contigs produced by today's assemblers have high precision. In our experiments, we use IDBA-UD [22], a non-aggressive and accurate assembler that is applicable for metagenomic data. Thus, large and accurate initial clusters can be obtained which can improve the performance of merging clusters. Each read that cannot be aligned to any contig will be treated as a cluster by itself initially and be merged based on *w*-mer sharing probability.

After merging, each group of reads (and contigs) will represent a *virtual contig* of a genome in the sense that these overlapping reads cover 'multiple' fragments of the same genome but these fragments might not be able to form a single contig in the usual way because they might be disconnected (grouped together based on paired-end reads) or contain repeated regions (branches which usually break up contigs). Thus, the 'length' (estimated based on the number of reads) of a virtual contig can be much longer than a contig and this facilitates later annotation.

### Phase 2: q-mer distribution estimation and clustering

Observation (C) suggests that contigs or virtual contigs that share similar *q*-mer distributions have higher probability of being sampled from the same genome. Thus, virtual contigs produced in the first phase should be further clustered based on *q*-mer distributions. However, as the number of reads sampled from different regions of a genome can vary due to sequencing bias, the *q*-mer distributions of a virtual contig cannot be estimated directly from the *q*-mer distributions of the reads in the virtual contig. We have to identify the overlapping regions of the reads and estimate the length of the virtual contigs as in [11].

Since the 'length' of the virtual contig produced by MetaCluster-TA is much longer than MetaCluster-5.0, *q*-mer distributions for larger *q* (*q* = 5 instead of 4) value can be estimated and better clustering results can be obtained. MetaCluster-TA groups virtual contigs using *q*-mers of different *q* values depending on their lengths. The 5-mer distributions of long virtual contigs (of length at least 10 k bp) are estimated and these virtual contigs are grouped using the *K*-means clustering algorithm based on Spearman distances of their 5-mer distributions. Short virtual contigs (of length less than 10 k bp) are assigned to their nearest clusters based on the Spearman distances of their 4-mer distributions. Note that only long virtual contigs based on their 5-mer distributions are used in clustering because short virtual contigs preserve less information and they may become noisy for the clustering, thus resulting in inaccurate clusters.

### Phase 3: cluster annotation and merging

In this phase, we assign taxonomy to each cluster. For each cluster, we align each contig to the reference genome using BLASTN and find the genome with the highest alignment score. The cluster will then be annotated to the lowest common ancestor of these aligned genomes using MEGAN4, i.e. all reads and contigs in the cluster will be annotated to the LCA of all aligned genomes. When more than one cluster being annotated to the same species (or lower taxonomy), the clusters will be merged for a better binning result. However, when two clusters be annotated to the same genus (or higher taxonomy), the clusters will not be merged as they may represents reads sampled from different species from the same genus.

### Time complexity

Phases 1 and 2, like MetaCluster 4.0 and 5.0, take a reasonably long time. Phase 3 aligns contigs in cluster using BLASTN with time complexity O(*n*), where *n* is the total length of contigs. The annotation step of aligning each read to known genomes, based on BLASTN, is the bottleneck for MEGAN4, which takes an extremely long time. However, our annotation step annotates virtual contigs instead of reads/contigs, which is much more efficient.
